# Rehabilitation with implant-supported overdentures in total edentulous patients: A review

**DOI:** 10.4317/jced.50817

**Published:** 2013-12-01

**Authors:** Juan F. Martínez–Lage-Azorín, Gustavo Segura-Andrés, Joan Faus-López, Rubén Agustín-Panadero

**Affiliations:** 1 Licenciado en Odontología por la Universidad de Murcia. Máster en Prótesis y Oclusión por la Universidad de Murcia. Máster de Estética, Escuela Superior de Implantología, Barcelona; 2 Licenciado en Odontología por la Universidad de Valencia; 3 Doctor en Odontología por la Universidad de Valencia. Director del Máster en Periodoncia y Osteointegración, Instituto Valenciano de Investigaciones Odontológicas (IVIO). Profesor de Periodoncia, Universidad CEU Cardenal Herrera, Valencia; 4 Licenciado en Odontología por la Universidad Complutense de Madrid. Máster en Prótesis Bucofacial. Universidad Complutense de Madrid. Profesor Asociado de la Unidad de Prostodoncia y Oclusión. Facultad de Medicina y Odontología. Universidad de Valencia

## Abstract

Objectives: The main aim of this review article is to discuss implant-supported overdentures (ISOs) as treatment in edentulous patients. Besides, we will try to discuss among the different treatment options in such patients and to analyze their validity when ISOs are compared with other clinical modalities. At the same time, we will try to suggest clinical guidelines supported by current clinical studies. Material and methods: We performed a Medline search and review of pertinent articles on the mentioned subject from 1986 to 2011. As a searching strategy, we used the following words: implant-supported overdentures, attachment systems, Locator attachment, cantilever, fixed prosthesis. Results and conclusions: Implant-supported overdentures constitute an accurate and predictable treatment option and achieve a higher patients’ satisfaction. This type of treatment constitutes a cheaper treatment than fixed prostheses and in some patients, with loss of lip support or with an interoclusal space larger than 15 mm, the choice of implant-supported overdentures seems to prevent future aesthetic or phonetic problems.

** Key words:**Overdentures, implant occlusion, implant rehabilitation, total edentulous rehabilitation, fixed prosthesis.

## Introduction

**Figure 1:**
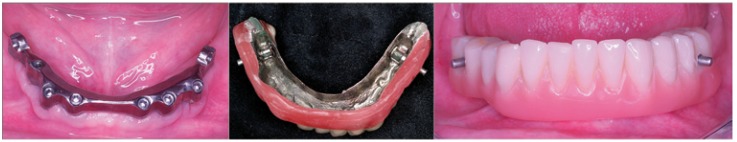
Photograph showing mandibular overdenture with six implants behaving as a fixed denture but with easier hygiene.

An implant-supported overdenture (ISO) is a removable complete denture combined with implants designed to improve stability in the oral environment. Depending on their support, we may classify them in: a) Implant-retained and mucous-supported overdentures, if the denture is buttressed by tissues and are retained on the implants, and b) Implant-retained and supported overdenture, if support and retention are due to the implants that behave as a fixed denture but the patient can remove it for an adequate oral hygiene (Fig. 1).

ISOs constitute a good management choice when edentulous patients are unsatisfied with conventional complete dentures because ISOs afford greater retention, support and stability. Also, ISOs seems to be indicated in patients who cannot afford a fixed ISO or have anatomic limitations to implants or who have phonetic-aesthetic problems as loss of lip support, very long clinical crowns, or wide interproximal spaces.

As ISOs are removable dentures, their hygiene is very easy and although they would need often control appointments for maintenance, they have a great acceptance by the patients.

A) Selecting an adequate ISO attachment.

Clinicians have selected different attachment systems based on factors such as durability, patient demand, cost effectiveness, technical simplicity, and retention (1). Attachments can be classified depending on its function as a) rigid, if they do not allow any denture dislodgements, or b) resilient, when they allow translation, rotation, axial or hinge over posterior axes movements or a combination of them because of their flexibility. With rigid attachments, the implant will receive 100% of occlusal load, whilst, with resilient attachments, occlusal load will be supported by implant, denture or fibromucous. Currently, the most used attachments are:

1.“O” Ring or Ball attachment. Ball attachment are considered the simplest type of attachment for clinical application with tooth or implant supported overdentures. It has a screw-retained male abutment in the implant with a spherical shape on its occlusal portion, and a prosthetic anchored female part that can be metallic or covered with nylon having a different retention range. These attachment do not need a great prosthetic space and they allow hinge and rotation dislodgements. However, the specific design of the ball attachment may influence the amount of free movement thereby limiting its resiliency (2). However, these attachments cannot be used with non-parallel implants.

2. Magnetic attachments. Basically, they consist of one magnet attached to the denture and another to the implant. They constitute a simple and comfortable system for the patient as magnet attraction guides the denture insertion. On the other hand, they have a weaker lateral stability and retention in comparison with mechanic attachments as ball or bar devices. In addition, they are susceptible to corrosion by saliva, explaining why they are clinically less often used. However, a new generation of rare-earth magnetic attachments could improve their properties and be clinically more often utilized (3). These new attachments may still be a useful treatment option for edentulous patient with weak muscle disease such as Parkinson’s disease patients, because they not only keep the denture stable, but also need less force to insert and remove the denture (4).

3. Bar attachments. Bar constitute an excellent anchorage system that provides greater retention, enabling better force balance by its splinting effect and it can also correct severe unparalellisms. The retention elements or clips are interchangeable and can be reactivated. The main disadvantages of bar attachments are the need for a large prosthetic space and the risk of mucositis due to an inadequate oral hygiene under the bar. Bars need to be parallel to the rotation axis, be straight and be positioned 1-2 mm to the alveolar crest. There are some different bar designs as Ackermann Bar (spherical shape), Dolder Bar (ovoid or “U” shape) and Hader Bar (keyhole shape).Also, there are implant-supported milled bars overdentures. They are bars with precision attachments and rigid anchorage, made by casting, electroerosion or CAD-CAM. They need a larger prosthetic space because of its volume and necessitate a good implant anchorage to support functional forces. They have double retention: by wall convergence of two degrees and by using other attachments systems anchored to the bar as Locator® (Zest Anchors Inc., Escondido, EEUU) or ball attachments.

4. Locator attachments. The male part consists of an implant screw-metallic abutment and the female part of a metallic cap lined with nylon of different colors depending on their retention capacity, which is anchored to the denture. There are two types of nylon: a) those with internal and external retention for well-positioned implants (from more to less retention: transparent, pink, blue) and b) with external retention for parallel implants (from more to less retention: green, orange, red). Finally, there is a yellow nylon for laboratory use that can also be used as temporary nylon. These attachments do not need a large prosthetic space and they can correct unparalellism up to 40 degrees. The attachments allows for rotation dislodgement and their utilization is widely endorsed in the current literature.

5. Other attachments. Ceka Attachment, ERA Attachment, VKS-OC RS Attachment, etc.

B) Selecting an adequate retention system.

**Figure 2:**
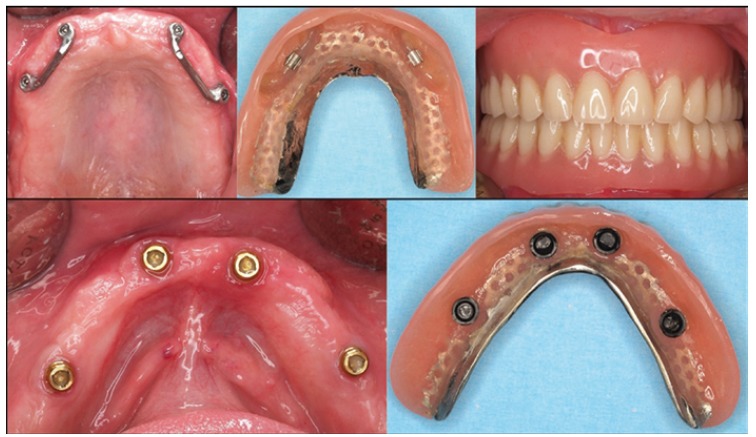
Photograph showing mandibular overdenture with six implants behaving as a fixed denture but with easier hygiene.

1. Depending on upper and lower jaw: in the mandible it will be easier to place parallel implants, thus, ball or Locator attachments would be indicated. In the maxillary, implants divergent emergency, worse bone quality and the use of short implants due to sinus proximity, will mandate the use of bar attachments (Fig. 2).

2. Depending on the arch form: bar attachments will be indicated in wide arches. On the other hand, in narrow arches using ball or Locator attachments would be indicated.

3. Depending on bone reabsorption rate and implants’ length: if implant is at least 10 mm long, it can be used as unsplinted, but if it less than 10 mm long , it will be indicated that the implant be splinted with bar attachments. According to Jemt and Lekholm (5), there were more failures (24%) in implants less than 10 mm long.

4. Depending on implant location: if implants are placed quite far from each other, it will not be indicated to use bar attachments due to increase of bone stress.

C. Selecting an adequate cantilever.

Finally, it is important to consider cantilevers because their presence is associated with a larger overload of distal implant if overdenture base does not adjust perfectly to the mucous (6). A cantilever should be shorter than the distance between implants to avoid overloads. Dunnen et al. (7), in a comparative study among structures with or without cantilevers, found a higher fracture bar rate in those with cantilevers, and, also, the fractures were localized at the cantilever level. In addition, it is advisable to avoid the placement of clips on the cantilever as this will increase tensions of the adjacent implant.

Semper et al. (8) concluded that a cantilever in the mandible shorter than 12 mm does not involve a great bone reabsorption, whilst, on the other hand, maxillary treatments are more susceptible to fail.

## Material and methods

We performed a Medline search and review of pertinent articles on the subject in a period from 1986 to 2011. The searching strategy included next keywords “implant-supported overdentures”, “attachment systems”, “Locator attachment”, “magnetic attachment”, “cantilever” and “fixed prosthesis”. In addition of the electronic searching, we did a manual searching with the registered literature references and dental journals. After a selection process, we have included some comparative studies based on the efficacy of different attachment systems and also, clinical studies based on the survival of prosthesis restorations with different attachment systems. We also include some illustrations of clinical cases.

## Results and Discussion

A) Maxillary Treatment Choices.

**Figure 3:**
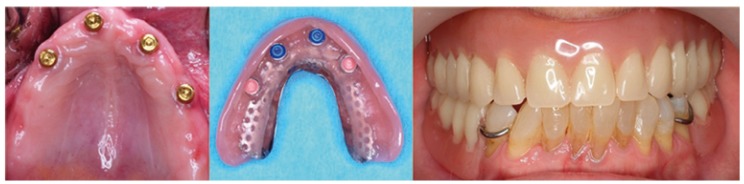
Photograph illustrating maxillar overdenture with four implants and Locator´s attachments.

ISO will be indicated in severe bone reabsorption, as it might compensate the loss of lip support avoiding air or saliva lost when speaking as it often occurs with fixed implant rehabilitations. Due to biomechanical requirements and worse bone quality, treatment options are just two: four or six-implant-supported overdentures, with an antero-posterior extension as wider as possible. Preferably, implants should be splinted with a bar without cantilevers that follow the arch shape to avoid fractures. On the other hand, there are some clinicians who prefer using a Locator system (Fig. 3), although this fact is less documented in the literature. Slot et al. (9), in a meta-analysis to evaluate the most successful maxillary treatment, concluded that six implants and a bar followed by four implants and a bar and last, four implants and ball attachments, constitute the most successful treatment

B) Mandibular Treatment Choices.

If there are good or excellent anatomical conditions with an inverted “U” shape alveolar ridge, without great bone reabsorptions, with support and lateral stability and basic patient demands, the ideal choice would be a splinted or unsplinted two-implants-supported overdenture. Implants should be parallel, perpendicular to the occlusal plane, and be situated at the same height to avoid faster wear in the highest or more leaning implant. According to Al-Ghafli et al. (10), implant angulation affects negatively the attachment retention longevity. In addition, implants should be placed equidistant to the midline to avoid instability, retainers wear, or abutment loosening. Finally, the distance between implants should be from 14 to 16 mm to avoid stress to the implants due to a long cantilever.

Another option would be to use a splinted three-implant-supported overdenture that will limit denture rotation dislodgement. Geckili et al. (11) in a 3-year follow-up study, of patients wearing mandibular three-implant-supported overdentures, found 100% of survival rate.

If there are severe or moderate anatomical conditions, with great bone posterior alveolar ridge reabsorptions and retention, support and stability loss, as well as high patient´s demand, it will be indicated to use a splinted or unsplinted four-implant-supported overdenture.

In 1986, Babbush et al. (12), in an 8-year follow-up study of edentulous patients treated with splinted four-implant-supported overdentures, reported an 88% survival rate. In 1997, Chiapasco et al. (13), in a 6-year follow-up study with 226 edentulous patients with same ISO as last, reported a 96.9% survival rate. In 2000, Gatti et al. (14) showed a survival rate over 96%.

In 2011, Burns et al. (15) concluded that the greatest retention was found with four splinted implants with a bar although patients show a higher satisfaction with ball attachments in a survey of 30 patients treated with four-implant-supported overdenture and ball or bar attachments.

ISOs with five or more implants will be indicated on fixed implant-supported rehabilitation, although there are some clinicians who might use these types of overdentures in square-shaped arches.

Comparative studies by Rashid et al. (16) and Assunção et al. (17), in patients wearing conventional dentures and ISOs, they concluded that: ISOs produced less bone reabsorption, had greater retention and stability and that they possess a better chewing function, thus increasing patients’ satisfaction and improving their quality of life.

Ueda et al. (18), performed a 24-years follow-up study in patients wearing a mandibular ISO with bar or ball attachments, obtaining 85.9% of survival rate and concluded that ISOs constitute a long-term success treatment.

- Comparative studies.

(a) Studies based on retention, support and stability: In an in vitro study, Sadig et al. (19) concluded that Locator attachments had greater retention and stability than ball or magnetic attachments. Van Kampen et al. (20) also argued that magnetic attachments had a weaker retention and needed more maintenance than ball or bar attachments. Ceruti et al. (3) showed high satisfaction rates in a 1-year follow-up study of 17 patients wearing mandibular ISO with new generation rare-earth magnetic attachments, with less corrosion and longer retention, and who previously were wearing ISO with ball or bar attachments. These authors stated that these devices constitute a predictable treatment alternative when the patient is unhappy with ISO involving mechanic attachments. Weinländer et al. (21), in a comparative study among 76 patients wearing a mandibular ISO, some with resilient ovoid bar and some with rigid milled bar over four implants, concluded that milled bars required less maintenance. As well, Krennmair et al. (22), in a 5 year follow-up study among 51 patients wearing same mandibular ISOs, arrived at the same conclusions. According to Bueno-Samper et al. (23), milled bars constitute a predictable treatment alternative due to their retention and stability as a fixed denture and have the advantages of a removable denture.

(b) Studies based on prosthesis maintenance: According to Kleis et al. (24), Locator attachments need a greater maintenance due to their progressive loss of retention. On the other hand, Cakarer et al. (25) claimed that Locator attachments show less complications and that they possess better maintenance outcomes than ball or bar attachments.

(c) Studies based on marginal bone loss: Menicucci et al. (26), in a comparative study with different attachment systems, reported that bar attachments produced a greater marginal bone stress than ball attachments. Vercruyssen et al. (6), in a 16-year follow-up study of 459 patients wearing two-implant-supported mandibular overdenture, concluded that independently of the attachment type, the amount of bone loss was less than 0.1 mm per year and that the implants that suffered a larger marginal bone loss or failed (2.5%) that they attributed to other factors as smoking.

## Conclusions

Edentulous patients often do not get used to wear conventional dentures. Their support is compromised by progressive bone reabsorption that will increase patients’ instability, insecurity and discomfort. Overdentures constitute a predictable and secure therapeutic alternative affording a great patient´s satisfaction due to simpler hygiene and good chewing efficiency (27). Overdenture use represents a cheaper treatment than fixed prostheses and, in some cases as those with lip support loss or with an interocclusal space larger than 15 mm, their use will prevent future aesthetic or phonetic problems. In the maxillary, implant divergent emergency, worse bone quality and the use of short implants due to anatomical limits as sinus, will condition the use of bar attachments. On the other hand, in the mandible, it will be easier to place parallel implants, thus we might use Locator or ball systems that will help to maintain a correct hygiene.

## References

[1] Lee DJ (2013). Performance of attachments used in implant-supported overdentures: review of trends in the literature. J Periodontal Implant Sci.

[2] John J, Rangarajan V, Savadi RC, Satheesh Kumar KS, Satheesh Kumar P (2012). A finite element analysis of stress distribution in the bone, around the implant supporting a mandibular overdenture with ball/o ring and magnetic attachment. J Indian Prosthodont Soc.

[3] Ceruti P, Bryant SR, Lee JH, MacEntee MI (2010). Magnet-retained implant-supported overdentures: review and 1-year clinical report. J Can Dent Assoc.

[4] Kim HY, Lee JY, Shin SW, Bryant SR (2012). Attachment systems for mandibular implant overdentures: a systematic review. J Adv Prosthodont.

[5] Jemt T, Lekholm U (1995). Implant treatment in edentulous maxillae: a 5-year follow-up report on patients with different degrees of jaw resorption. Int J Oral Maxillofac Implants.

[6] Vercruyssen M, Quirynen M (2010). Long-term, retrospective evaluation (implant and patient-centered outcome) of the two-implant-supported overdenture in the mandible. Part 2: marginal bone loss. Clin Oral Implants Res.

[7] den Dunnen AC, Slagter AP, de Baat C, Kalk W (1998). Adjustments and complications of mandibular overdentures retained by four implants. A comparison between superstructures with and without cantilever extensions. Int J Prosthodont.

[8] Semper W, Heberer S, Nelson K (2010). Retrospective analysis of bar-retained dentures with cantilever extension: marginal bone level changes around dental implants over time. Int J Oral Maxillofac Implants.

[9] Slot W, Raghoebar GM, Vissink A, Huddleston Slater JJ, Meijer HJ (2010). A systematic review of implant-supported maxillary overdentures after a mean observation period of at least 1 year. J Clin Periodontol.

[10] Al-Ghafli SA, Michalakis KX, Hirayama H, Kang K (2009). The in vitro effect of different implant angulations and cyclic dislodgement on the retentive properties of an overdenture attachment system. J Prosthet Dent.

[11] Geckili O, Bilhan H, Mumcu E (2011). Clinical and radiographic evaluation of three-implant-retained mandibular overdentures: a 3-year retrospective study. Quintessence Int.

[12] Babbush CA, Kent J, Misiek D (1986). Titanium plasma-sprayed (TPS) screw implants for the reconstruction of the edentulous mandible. J Oral Maxillofac Surg.

[13] Chiapasco M, Gatti C, Rossi E, Haefliger W, Markwalder TH (1997). Implant-retained mandibular overdentures with immediate loading. A retrospective multicenter study on 226 consecutive cases. Clin Oral Implants Res.

[14] Gatti C, Haefliger W, Chiapasco M (2000). Implant-retained mandibular overdentures with immediate loading: a prospective study of ITI implants. Int J Oral Maxillofac Implants.

[15] Burns DR, Unger JW, Coffey JP, Waldrop TC, Elswick RK (2011). Randomized, prospective, clinical evaluation of prosthodontic modalities for mandibular implant overdenture treatment. J Prosthet Dent.

[16] Rashid F, Awad MA, Thomason JM, Piovano A, Spielberg GP, Scilingo E (2011). The effectiveness of 2-implant overdentures - a pragmatic international multicentre study. J Oral Rehabil.

[17] Assunção WG, Barão VA, Delben JA, Gomes EA, Tabata LF (2010). A comparison of patient satisfaction between treatment with conventional complete dentures and overdentures in the elderly: a literature review. Gerodontology.

[18] Ueda T, Kremer U, Katsoulis J, Mericske-Stern R (2011). Long-term results of mandibular implants supporting an overdenture: implant survival, failures, and crestal bone level changes. Int J Oral Maxillofac Implants.

[19] Sadig W (2009). A comparative in vitro study on the retention and stability of implant-supported overdentures. Quintessence Int.

[20] Van Kampen F, Cune M, van der Bilt A, Bosman F (2003). Retention and postinsertion maintenance of bar-clip, ball and magnet attachments in mandibular implant overdenture treatment: an in vivo comparison after 3 months of function. Clin Oral Implants Res.

[21] Weinländer M, Piehslinger E, Krennmair G (2010). Removable implant-prosthodontic rehabilitation of the edentulous mandible: five-year results of different prosthetic anchorage concepts. Int J Oral Maxillofac Implants.

[22] Krennmair G, Krainhöfner M, Piehslinger E (2008). The influence of bar design (round versus milled bar) on prosthodontic maintenance of mandibular overdentures supported by 4 implants: a 5-year prospective study. Int J Prosthodont.

[23] Bueno-Samper A, Hernández-Aliaga M, Calvo-Guirado JL (2010). The implantsupported milled bar overdenture: A literature review. Med Oral Patol Oral Cir Bucal.

[24] Kleis WK, Kämmerer PW, Hartmann S, Al-Nawas B, Wagner W (2010). A comparison of three different attachment systems for mandibular two-implant overdentures: one-year report. Clin Implant Dent Relat Res.

[25] Cakarer S, Can T, Yaltirik M, Keskin C (2011). Complications associated with the ball, bar and Locator attachments for implant-supported overdentures. Med Oral Patol Oral Cir Bucal.

[26] Menicucci G, Lorenzetti M, Pera P, Preti G (1998). Mandibular implant-retained overdenture: finite element analysis of two anchorage systems. Int J Oral Maxillofac Implants.

[27] Feine JS, de Grandmont P, Boudrias P, Brien N, LaMarche C, Taché R (1994). Within-subject comparisons of implant-supported mandibular prostheses: choice of prosthesis. J Dent Res.

[28] Sadowsky SJ, Caputo AA (2000). Effect of anchorage systems and extension base contact on load transfer with mandibular implant-retained overdentures. J Prosthet Dent.

